# The Episode of National Lockdown in the Pandemic: Air Traffic Restriction as the Control Strategy and Its Impact on Existing Cases and Recovery Rate of Novel Coronavirus Disease in Megacities of China

**DOI:** 10.1017/dmp.2020.294

**Published:** 2020-08-12

**Authors:** Jiannan Li, Zhaoguo Wang, Bocong Yuan, Haixuan Liang, Wenqi Liang

**Affiliations:** International School of Business & Finance, Sun Yat-sen University, Guangzhou, China; School of Economics and Management, Shenyang Agricultural University, Shenyang, China; School of Tourism Management, Sun Yat-sen University, Guangzhou, China

**Keywords:** air traffic restriction, COVID-19, existing cases, megacity, recovery rate

## Abstract

**Objective:**

The effectiveness of air traffic restriction in containing the spread of infectious diseases is full of controversy in prior literature. In January 2020, the Civil Aviation Administration of China (CAAC) announced air traffic restriction in response to the coronavirus disease (COVID-19) pandemic. This study’s aim is to empirically examine the policy effectiveness.

**Method:**

The data from 2 third-party platforms are used in this investigation. The COVID-19 data from the platform DXY and the air traffic data from Airsavvi are matched to each other. The robust panel regression with controlling city effect and time effect is conducted.

**Results:**

The curvilinear relations are found between the air traffic restriction and the existing cases, and the recovery rate (quadratic term = 9.006 and −0.967, respectively). As the strength of air traffic restriction is growing, the negative effect (-8.146) of air traffic restriction on the existing cases and the positive effect (0.961) of air traffic restriction on the recovery rate, respectively, begin decreasing.

**Conclusion:**

On the macro level, the air traffic restriction may help alleviate the growth of existing cases and help raise the recovery rate of COVID-19 in megacities of China, but both these effects will marginally recede as the restriction strength is intensifying.

The air traffic network may turn to be the network of infectious disease propagation during the pandemic. The transmission risk of infectious diseases lies not only in the post-flight facilitation of population mobility across cities, but also in the inflight facilitation of virus transmission in the cabin of a single-aisle aircraft or the economy class.^1,2^ During the outbreak of coronavirus disease (COVID-19) in China, substantial medical resources used in clinical treatment have to be reallocated to the prevention of potential transmission risk in public transport facilities. Passengers of the same flight would be quarantined and arranged for the rigorous detection of virus nucleic acid testing, once a certain passenger is found to have respiratory symptoms or fever in this flight. Thus, the risk of inflowed suspected cases may be amplified for their mutual long-time sharing of the space during the flight, and the above stringent arrangement would inevitably deteriorate the shortage of medical resource. Besides, the fast and massive spread of the virus via air traffic brings an additional burden on the health system, which can also lead to a reduction in the recovery rate. On January 23 and 24, 2020, the Civil Aviation Administration of China (CAAC) makes responses to the pandemic emergency and announces the temporary regulation of air traffic restriction.^3-5^ Airline companies are urged to keep a close watch on the development of COVID-19 in Hubei and to reduce the number of flights.^3-5^

However, prior studies show controversies on the effectiveness of air traffic restriction in the containment of pandemic development. Accessing public transport within 5 days of symptom onset is reported to be associated with a sixfold additional risk of consulting for acute respiratory infection.^6^ The strengthening of air traffic restriction is encouraged as it can break down the inter-regional transmission dynamics.^7^ On the other side, some researchers place suspicion on the effectiveness of air traffic restriction in containing the pandemic. For instance, the influence of air traffic control on the epidemic development during Mexico’s 2009 H1N1 pandemic is almost negligible in the epicenter area, and this control scarcely curbs but only delays for a few days of the spread in other areas.^8^ Similar results are reached in an Ebola virus research proposed by Polletto et al.^9^ A most recent evaluation about the air traffic restriction as the control strategy against COVID-19 spread between China and Japan identifies just a very tiny delay of the epidemic outbreak, and authors suggest that the decision to restrict air traffic should be balanced between the estimated epidemic impact and forthcoming economic fallout, as the benefit of delay is quite small.^10^ To clarify previous controversies by adding new empirical evidence, this study’s aim is to examine whether this regulation can affect the existing cases and recovery rate of COVID-19 in China.

## METHODS

Multisource data from the platform DXY (the real-time COVID-19 data can be accessed on https://ncov.dxy.cn/ncovh5/view/pneumonia) and from the airline data platform Airsavvi (a brief form of dataset can be accessed on http://covid.airsavvi.com/) are matched to each other and used in this study. Chinese megacities whose airports have passenger throughput of over 30 million a year are included in the sample (ie, Beijing, Shanghai [both Pudong and Hongqiao Airports are included], Guangzhou, Chengdu, Shenzhen, Kunming, Xi’an, Chongqing, Hangzhou, Nanjing). The time span of data is from January 23, 2020, to March 18, 2020. The panel data set is unbalanced given that the available data of different cities may have different time spans. The outcome variable “recovery rate” for each megacity is calculated by, *recovery rate = cured cases/confirmed cases*. Besides, the “existing case” is calculated by, *confirmed cases + suspected cases − death cases − cured cases*.

The independent variable “air traffic restriction” is defined as follows:
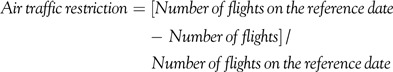



In the above equation, the reference date is during January 6, 2020 (Monday) to January 12, 2020 (Sunday), given that this time span is before the official announcement of air traffic restriction. For instance, the air traffic restriction on February 5, 2020 (Wednesday) is calculated as “the difference between the number of flights on February 5, 2020 (Wednesday) and January 8, 2020 (Wednesday)” divided by the number of flights on January 8, 2020 (Wednesday). The regression is conducted as follows:
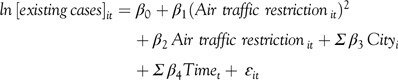


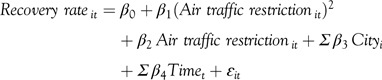



In which, the subscript *i* indicates the *i*-th city and the subscript *t* indicates the *t*-th day.

## RESULTS

As indicated in Table 1, the coefficients of *air traffic restriction-square* (referring to the quadratic term of air traffic restriction) are significant. Consistent with the common practice of previous studies,^11^ the significant quadratic term implies that there is a U-shape curve between variables, and the positive (or negative) sign of the quadratic term indicates an upward (or downward, also called *inverted*) U-shape curve. Besides, the quadratic term means that, with the independent variable approaching to the inflection point, the rate of change in the dependent variable becomes increasingly smaller. The U-shape curves found in this study are the ones without the inflection point but infinitely approaching it.

**TABLE 1:** The Effect of Air Traffic Restriction on the Existing Cases and Recovery Rate of COVID-19

	Panel A. Outcome Variable ln Existing Cases				Panel B. Outcome Variable Recovery Rate			
	Coef	SE	*P*-value	95% CI	Coef	SE	*P*-value	95% CI
Independent Variable								
Air traffic restriction	−8.146	0.745	0.000	[−9.606, −6.687]	0.961	0.112	0.000	[0.742, 1.181]
[Air traffic restriction] square	9.006	0.914	0.000	[7.215, 10.797]	−0.967	0.137	0.000	[−1.235, −0.698]
City Effect								
*Wuhan*	Reference				Reference			
*Beijing*	−2.101	0.363	0.000	[−2.812, −1.390]	−0.061	0.054	0.260	[−0.167, 0.045]
*Shanghai*	−2.668	0.382	0.000	[−3.416, −1.919]	0.048	0.057	0.396	[−0.063, 0.160]
*Guangzhou*	−2.635	0.383	0.000	[−3.386, −1.883]	−0.001	0.057	0.980	[−0.113, 0.111]
*Chengdu*	−3.117	0.391	0.000	[−3.882, −2.351]	−0.024	0.058	0.682	[−0.138, 0.090]
*Shenzhen*	−2.379	0.385	0.000	[−3.133, −1.625]	0.001	0.057	0.989	[−0.112, 0.113]
*Kunming*	−4.526	0.369	0.000	[−5.250, −3.802]	0.006	0.055	0.919	[−0.103, 0.114]
*Xi’an*	−3.858	0.348	0.000	[−4.540, −3.175]	−0.008	0.052	0.871	[−0.110, 0.093]
*Chongqing*	−2.137	0.376	0.000	[−2.874, −1.400]	−0.005	0.056	0.924	[−0.115, 0.105]
*Hangzhou*	−3.699	0.362	0.000	[−4.409, −2.989]	0.079	0.054	0.145	[−0.027, 0.185]
*Nanjing*	−4.150	0.358	0.000	[−4.853, −3.448]	0.039	0.053	0.463	[−0.065, 0.143]
Time Effect								
*2020/1/23*	Reference				Reference			
*2020/1/24*	0.510	0.337	0.130	[−0.150, 1.171]	−0.053	0.051	0.298	[−0.152, 0.047]
*2020/1/25*	1.122	0.339	0.001	[0.457, 1.788]	−0.072	0.051	0.158	[−0.172, 0.028]
*2020/1/26*	1.448	0.339	0.000	[0.784, 2.111]	−0.072	0.051	0.159	[−0.172, 0.028]
*2020/1/27*	1.472	0.334	0.000	[0.817, 2.127]	−0.035	0.050	0.490	[−0.133, 0.064]
*2020/1/28*	2.099	0.339	0.000	[1.434, 2.764]	−0.074	0.051	0.147	[−0.174, 0.026]
*2020/1/29*	2.485	0.342	0.000	[1.815, 3.155]	−0.089	0.051	0.084	[−0.190, 0.012]
*2020/1/30*	2.795	0.344	0.000	[2.121, 3.469]	−0.099	0.052	0.056	[−0.200, 0.002]
*2020/1/31*	3.429	0.356	0.000	[2.731, 4.128]	−0.143	0.054	0.008	[−0.248, −0.038]
*2020/2/1*	3.935	0.369	0.000	[3.211, 4.659]	−0.179	0.056	0.001	[−0.288, −0.070]
*2020/2/2*	4.161	0.374	0.000	[3.429, 4.893]	−0.187	0.056	0.001	[−0.297, −0.076]
*2020/2/3*	4.373	0.379	0.000	[3.629, 5.116]	−0.200	0.057	0.000	[−0.312, −0.089]
*2020/2/4*	4.665	0.392	0.000	[3.897, 5.433]	−0.216	0.059	0.000	[−0.332, −0.101]
*2020/2/5*	4.679	0.390	0.000	[3.916, 5.443]	−0.180	0.059	0.002	[−0.295, −0.065]
*2020/2/6*	4.740	0.391	0.000	[3.973, 5.508]	−0.170	0.059	0.004	[−0.286, −0.055]
*2020/2/7*	4.850	0.391	0.000	[4.084, 5.616]	−0.148	0.059	0.012	[−0.263, −0.032]
*2020/2/8*	4.823	0.389	0.000	[4.061, 5.585]	−0.116	0.059	0.048	[−0.231, −0.001]
*2020/2/9*	4.847	0.386	0.000	[4.090, 5.605]	−0.095	0.058	0.103	[−0.209, 0.019]
*2020/2/10*	4.894	0.393	0.000	[4.125, 5.664]	−0.096	0.059	0.103	[−0.212, 0.019]
*2020/2/11*	4.771	0.397	0.000	[3.992, 5.549]	−0.079	0.060	0.189	[−0.196, 0.039]
*2020/2/12*	4.740	0.398	0.000	[3.960, 5.520]	−0.048	0.060	0.426	[−0.165, 0.070]
*2020/2/13*	4.710	0.400	0.000	[3.926, 5.493]	−0.040	0.060	0.504	[−0.158, 0.078]
*2020/2/14*	4.815	0.398	0.000	[4.035, 5.596]	−0.013	0.060	0.831	[−0.130, 0.105]
*2020/2/15*	4.733	0.399	0.000	[3.951, 5.515]	0.023	0.060	0.706	[−0.095, 0.140]
*2020/2/16*	4.852	0.397	0.000	[4.073, 5.631]	0.039	0.060	0.514	[−0.078, 0.156]
*2020/2/17*	4.654	0.399	0.000	[3.872, 5.435]	0.089	0.060	0.136	[−0.028, 0.207]
*2020/2/18*	4.548	0.400	0.000	[3.764, 5.333]	0.121	0.060	0.044	[0.003, 0.239]
*2020/2/19*	4.598	0.399	0.000	[3.816, 5.381]	0.148	0.060	0.014	[0.031, 0.266]
*2020/2/20*	4.497	0.399	0.000	[3.714, 5.280]	0.196	0.060	0.001	[0.079, 0.314]
*2020/2/21*	4.504	0.398	0.000	[3.724, 5.284]	0.238	0.060	0.000	[0.121, 0.356]
*2020/2/22*	4.350	0.399	0.000	[3.568, 5.132]	0.277	0.060	0.000	[0.160, 0.395]
*2020/2/23*	4.379	0.397	0.000	[3.601, 5.157]	0.300	0.060	0.000	[0.183, 0.417]
*2020/2/24*	4.255	0.397	0.000	[3.478, 5.032]	0.336	0.060	0.000	[0.219, 0.452]
*2020/2/25*	4.181	0.397	0.000	[3.402, 4.959]	0.353	0.060	0.000	[0.236, 0.470]
*2020/2/26*	4.080	0.396	0.000	[3.305, 4.856]	0.390	0.060	0.000	[0.274, 0.507]
*2020/2/27*	3.979	0.396	0.000	[3.202, 4.756]	0.410	0.060	0.000	[0.294, 0.527]
*2020/2/28*	3.956	0.395	0.000	[3.183, 4.730]	0.428	0.059	0.000	[0.312, 0.545]
*2020/3/4*	3.377	0.391	0.000	[2.612, 4.143]	0.541	0.059	0.000	[0.426, 0.656]
*2020/3/5*	3.219	0.393	0.000	[2.449, 3.990]	0.558	0.059	0.000	[0.442, 0.674]
*2020/3/6*	3.220	0.394	0.000	[2.448, 3.993]	0.578	0.059	0.000	[0.463, 0.694]
*2020/3/7*	3.014	0.398	0.000	[2.234, 3.794]	0.586	0.059	0.000	[0.469, 0.702]
*2020/3/8*	3.161	0.398	0.000	[2.381, 3.941]	0.598	0.059	0.000	[0.483, 0.714]
*2020/3/9*	3.029	0.398	0.000	[2.249, 3.809]	0.606	0.059	0.000	[0.490, 0.722]
*2020/3/10*	2.901	0.401	0.000	[2.116, 3.687]	0.612	0.059	0.000	[0.496, 0.728]
*2020/3/11*	2.801	0.397	0.000	[2.023, 3.579]	0.624	0.059	0.000	[0.509, 0.739]
*2020/3/12*	2.656	0.401	0.000	[1.871, 3.442]	0.633	0.059	0.000	[0.517, 0.750]
*2020/3/13*	2.499	0.399	0.000	[1.717, 3.280]	0.641	0.059	0.000	[0.525, 0.756]
*2020/3/17*	2.538	0.404	0.000	[1.746, 3.330]	0.659	0.059	0.000	[0.543, 0.774]
*2020/3/18*	2.669	0.408	0.000	[1.869, 3.468]	0.652	0.061	0.000	[0.532, 0.772]
Intercept	4.913	0.486	0.000	[3.960, 5.865]	0.049	0.073	0.502	[−0.094, 0.191]
Number of Observations	510				527			
Wald χ^2^ statistics	46.30 [*P*-value = 0.000]				85.10 [*P*-value = 0.000]			

As such, results of Table 1 show that the relations between air traffic restriction and the existing cases, and the recovery rate of COVID-19 are not linear. There is a U-shape relation between air traffic restriction and the existing cases of COVID-19 (coefficient of quadratic term = 9.006, *P* < 0.01; see Panel A), and the negative effect of air traffic restriction on the existing cases of COVID-19 (coefficient = −8.146, *P* < 0.01) marginally decreases as the strength of restriction is intensifying. In addition, there is an inverted U-shape relation between air traffic restriction and the recovery rate of COVID-19 (coefficient of quadratic term = −0.967, *P* < 0.01; see Panel B), and the positive effect of air traffic restriction on the recovery rate of COVID-19 (coefficient = 0.961, *P* < 0.01) begins decreasing as the strength of restriction is growing.

## DISCUSSION

On the macro level, air traffic restriction has a suppressing effect on the growth of existing cases of COVID-19 and plays a promoting role in the rise of recovery rate of COVID-19. However, these effects appear as not linear. With the strength of the air traffic restriction intensifying, the magnitude of these effects recedes gradually.

COVID-19 spreads quickly and the medical resources are insufficient to meet the demand for the growth spurt of treatment. At the same time, the timely arrangement for quarantine and virus nucleic acid testing are necessary for passengers on the same flight with suspected cases. The air traffic restriction may alleviate the shortage of medical resources and thus may help slow down the growth of existing cases and benefit the rise in the recovery rate. However, as the air traffic restriction intensifies to a certain degree, such beneficial effects will begin to marginally decrease. It implies that to raise the recovery rate and to control the growth of existing cases cannot depend solely on the air traffic limitation, but also on the increase in the supply of medical resources.

Since the real-time data regarding car, bus, and train are not available, the impact of land transportation restriction on the existing cases and recovery rate of COVID-19 cannot be examined. With the availability of real-time data of land transport, future research can make a comprehensive investigation on policy effectiveness of traffic restriction in containing the spread of infectious diseases. Further, there may become a tipping point where not only increasingly stringent restrictions do not improve recovery rates and reduce existing cases, but also actively begin to worsen them, since the increasingly stringent air travel restrictions may delay the supply of medical personnel and equipment/supplies. With the more comprehensive data available in the future, this point may also be identified in future research.

## CONCLUSION

During the COVID-19 pandemic in China, we observe the effective control over the infectious disease under the situation of prompt lockdown policy response (within 3 weeks since the Chinese Center for Disease Control and Prevention receives the report) and of full air traffic control nationwide. The delayed or insufficient shutdown of air traffic may dampen the effectiveness in pandemic control. As such, to cope well with potential pandemics in the future, it is necessary for the policy of air traffic restriction to consider the policy timeliness (how soon from the outbreak), policy strength (full or partially prohibited), geographic scale of the policy (regional or national), and so on. The discrepancy in aspects above for different cities may influence the overall effectiveness of the policy in infectious disease control.
